# Rubus crataegifolius Bunge regulates adipogenesis through Akt and inhibits high-fat diet-induced obesity in rats

**DOI:** 10.1186/s12986-016-0091-0

**Published:** 2016-04-27

**Authors:** Min-Sup Jung, Soo-Jung Lee, Yuno Song, Sun-Hee Jang, Wongi Min, Chung-Kil Won, Hong-Duck Kim, Tae Hoon Kim, Jae-Hyeon Cho

**Affiliations:** Institute of Animal Medicine, College of Veterinary Medicine, Gyeongsang National University, Jinju, 660-701 Korea; Department of Foods and Nutrition, Gyeongsang National University, Jinju, 660-701 Korea; Department of Environmental Health Science, New York Medical College, Valhalla, NY 10595 USA; Department of Food Science and Biotechnology, Daegu University, Gyeongsan, 38453 Korea; College of Veterinary Medicine, Gyeongsang National University, Jiju Daero 501, Jinju-city, Gyeongsangnamdo 660-701 Korea

**Keywords:** Rubus crataegifolius Bunge (RCB), 3 T3-L1 adipocyte, Adipogenesis, Akt, High fat diet, Obesity

## Abstract

**Background:**

Obesity is one of the greatest public health problems and major risk factors for serious metabolic diseases and significantly increases the risk of premature death. The aim of this study was to determine the inhibitory effects of Rubus crataegifolius Bunge (RCB) on adipocyte differentiation in 3 T3-L1 cells and its anti-obesity properties in high fat diet (HFD)-induced obese rats.

**Methods:**

3 T3-L1 adipocytes and HFD-induced obese rats were treated with RCB, and its effect on gene expression was analyzed using RT-PCR and Western blotting experiments.

**Results:**

RCB treatment significantly inhibited adipocyte differentiation by suppressing the expression of C/EBPβ, C/EBPα, and PPARγ in the 3 T3-L1 adipocytes. Subsequently, the expression of the PPARγ target genes aP2 and fatty acid synthase (FAS) decreased following RCB treatment during adipocyte differentiation. In uncovering the specific mechanism that mediates the effects of RCB, we demonstrated that the insulin-stimulated phosphorylation of Akt strongly decreased and that its downstream substrate phospho-GSK3β was downregulated following RCB treatment in the 3 T3-L1 adipocytes. Moreover, LY294002, an inhibitor of Akt phosphorylation, exerted stronger inhibitory effects on RCB-mediated suppression of adipocyte differentiation, leading to the inhibition of adipocyte differentiation through the downregulation of Akt signaling. An HFD-induced obesity rat model was used to determine the inhibitory effects of RCB on obesity. Body weight gain and fat accumulation in adipose tissue were significantly reduced by the supplementation of RCB. Moreover, RCB treatment caused a significant decrease in adipocyte size, associated with a decrease in epididymal fat weight. The serum total cholesterol (TC) and triglyceride (TG) levels decreased in response to RCB treatment, whereas HDL cholesterol (HDL-C) increased, indicating that RCB attenuated lipid accumulation in adipose tissue in HFD-induced obese rats.

**Conclusion:**

Our results demonstrate an inhibitory effect of RCB on adipogenesis through the reduction of the adipogenic factors PPARγ, C/EBPα, and phospho-Akt. RCB had a potent anti-obesity effect, reducing body weight gain in HFD-induced obese rats.

**Electronic supplementary material:**

The online version of this article (doi:10.1186/s12986-016-0091-0) contains supplementary material, which is available to authorized users.

## Background

Obesity arises from adipocyte hyperplasia and hypertrophy caused by the intake of excess energy [1]. Excess fat accumulates in adipocytes as excessive amounts of lipids (triglycerides), resulting in elevated triglyceride levels in plasma and in tissues such as liver and muscle, which leads to pathological dysfunction in these tissues [2, 3]. Thus, adipocyte hyperplasia may be an important factor in the development of obesity. To prevent obesity, it is important to maintain an adequate balance between energy accumulation and energy consumption.

The excessive accumulation of adipose tissue is caused by increased adipogenesis accompanied by adipocyte differentiation, which converts immature pre-adipocytes into adipocytes. Abnormal fat accumulation and adipocyte differentiation in adipose tissue are associated with the development of obesity [4]. Adipogenesis, the process of adipose cell development, is associated with multiple steps in the regulation of several transcription factors. During the differentiation process, many key transcriptional factors are involved, such as CCAAT-enhancer-binding protein (C/EBP)-δ and C/EBPβ, which can collectively induce the expression of peroxisome proliferator-activated receptor (PPAR)-γ and C/EBPα [5, 6]. PPARγ and C/EBPα also play important roles as major transcription factors in adipogenesis [5]. In addition, these transcription factors are involved in the acceleration of lipogenesis and lipid homeostasis by modulating the expression of target genes, such as adipocyte fatty acid-binding protein 2 (aP2) and fatty acid synthase (FAS) [7].

The intracellular signaling cascade involving insulin/phosphoinositide 3-kinase (PI3K)/Akt plays a major role in adipocyte differentiation. Akt phosphorylates and regulates a number of substrates that are involved in a diverse array of biological processes [8], and it is essential in the induction of PPARγ expression [9]. Pharmacological inhibition of the PI3K/Akt signaling pathway [10, 11] and dominant negative mutations [12] can abolish adipocyte differentiation by inhibiting PI3K/Akt activity. Moreover, the overexpression of constitutively active Akt increases glucose uptake and adipocyte differentiation in 3 T3-L1 adipocytes [13].

Rubus crataegifolius Bunge (RCB) is a type of red raspberry and is a member of the Rosaceae family. Rubus is one of the most diverse and largest genera in the Rosaceae family and is comprised of approximately 600 to 800 species, including blackberries, raspberries and their hybrids [14, 15]. Traditionally, Rubi coreanus (Rubi Fructus) fruit has been used as a medicinal agent for the treatment of impotence, spermatorrhea, enuresis, and asthma [16]. Rubi crataegifolius has also been used for treatment of rheumatic arthritis, hepatitis and lung cancer in China [17]. Traditional herbal medicines may have some potential to prevent obesity. Although the use of these fruits in fresh and processed food items, such as jams, jellies and juices, represents a multimillion dollar industry [18], their medicinal properties—including their anti-inflammatory effects on ulcers and their capacity to reduce blood cholesterol levels [19] and cell proliferation [20] in animal models—have recently attracted the attention of the international market. However, no reports documenting the anti-obesity effects of RCB have been published.

In this study, we hypothesized that RCB could exert the inhibitory effect on the differentiation of 3 T3-L1 preadipocytes and its in vivo inhibition of high fat diet (HFD)-induced obesity in rats. To study this possibility, the inhibitory effects of RCB on differentiation of 3 T3-L1 preadipocytes into adipocytes was examined by measuring triglyceride accumulation levels and expression levels of genes involved in adipogenesis and lipogenesis in adipocytes. We also investigated the underlying mechanism whether Akt signaling is critical for the anti-obesity function of RCB. Moreover, we examined the effects of RCB on body weight, adipose tissue mass, and lipid metabolism in HFD-induced obese rats.

## Methods

### Preparation of Rubus crataegifolius Bunge (RCB) extracts

The fresh fruits of *Rubus crataegifolius Bunge* (RCB) were collected in Gyeongsan-si, Gyeongsangbuk-do, Korea in September 2013. A voucher specimen representing this collection was identified by Prof. Gon-Sup Kim and deposited at the Animal Bio Resources Bank of Gyeongsang National University. The fresh fruits of RCB were prepared by alcohol extraction. The fruit bodies were peeled off and air-dried in an oven at an initial temperature of 30 °C, which was increased by 5 °C every 3 h until it reached 40 °C. The dried fruit bodies of RCB were milled into a powder (40 mesh). Fifteen grams of RCB powder were then suspended in an 80 % (v/v) ethanol solution using a mixer, followed by the extraction of the samples for 3 days with vigorous shaking at room temperature and filtering through Whatman No. 1 filter paper. The 80 % ethanol extracts of RCB were concentrated using rotary-vacuum evaporation at 50 °C and then freeze-dried. The RCB extract was stored in the freezer until it was used for experiments.

### Measurement of total phenolic content using folin-ciocalteu assay

The total phenolic content of RCB was measured using a spectrophotometer according to the Folin-Ciocalteu colorimetric method as previously described [21, 22]. Because quercetin is one of the polyphenol compounds found in RCB, the total phenolic content of the ethanol extract of RCB was expressed as mg catechin equivalents (QE)/g. Catechin was purchased from Sigma-Aldrich (St. Louis, MO. USA). The measurements were performed four times.

### Measurement of total flavonoids

Total flavonoid content was determined as previously described [23] with slight modifications. Briefly, 0.25 mL of RCB (100 μg/mL) was added to a tube containing 1 mL of double-distilled water. Following this, 0.075 mL of 5 % NaNO_2_, 0.075 mL of 10 % AlCl_3_ and 0.5 mL of 1 M NaOH were added sequentially at 0, 5 and 6 min. Finally, the volume of the reacting solution was adjusted to 2.5 mL with double-distilled water. The 410 nm absorbance of the solution was detected using an Ultrospec 2100 Pro Spectrophotometer (Section 3.3). The results are expressed in mg quercetin equivalents (QE)/g. The experiments were carried out in quadruplicate.

### Measurement of free radical scavenging activity using a 2,2-diphenyl-1-picrylhydrazyl (DPPH) assay

The free radical scavenging activity of RCB (100 μg/mL in DW) was measured using a method created by Brand-Williams [24] with slight modifications. The inhibition percentage was calculated using the following equation: Inhibition % = [(absorbance of control - absorbance of sample)/absorbance of control] × 100. The absorbance was measured using a spectrophotometer (Ultrospec 2100 pro; Amersham Pharmacia Biotech Co., Piscataway, NJ, USA). The experiments were carried out in quadruplicate.

### Measurement of superoxide anion (O2^●−^) radical scavenging and hydroxyl (OH^●^) radical scavenging activity

Superoxide radicals were generated according to a method described in a previous paper [25]. The samples (100 μg/mL in DMSO) were added to a reaction solution containing 100 μL of 30 mM EDTA (pH 7.4), 10 μL of 30 mM hypoxanthine in 50 mM NaOH, and 200 μL of 1.42 mM nitroblue tetrazolium (NBT). After the solution was preincubated at room temperature for 3 min, 100 μL of 0.5 U/mL xanthine oxidase was added to the mixture, and the volume was brought up to 3 mL with 50 mM phosphate buffer (pH 7.4). After the solution was incubated at room temperature for 20 min, absorbance was measured at 560 nm. The reaction mixture without xanthine oxidase was used as a blank (A1). The samples (A2) were added to the reaction mixture, in which O2^●−^ was scavenged and thereby inhibited the reduction of NBT. The absorbance was measured, and a decrease in O2^●−^ was represented by A2-A1. The scavenging activity of the superoxide anion radical (SRSA) was calculated using the following equation: SRSA % = (A2 − A1/A1) × 100. The scavenging activity of the samples (100 μg/mL) in DMSO on the hydroxyl radical (OH^●^) was measured using the deoxyribose method [26] with slight modifications. The deoxyribose assay was performed in 10 mM phosphate buffer (pH 7.4) containing 2.5 mM deoxyribose, 1.5 mM H_2_O_2_, 100 μM FeCl_3_, 104 μM EDTA, and a test sample (0.5 mg/mL). The reaction was initiated by adding ascorbic acid to a final concentration of 100 μM. The reaction mixture was incubated for 1 h at 37 °C in a water bath. After incubation, the color was developed by the addition of 0.5 % thiobarbituric acid followed by an addition of ice-cold 2.8 % trichloroacetic acid in 25 mM NaOH and incubation for 30 min at 80 °C. A control experiment was performed without the samples (A1). The samples (A2) were cooled on ice, and the absorbance was measured at 532 nm. The hydroxyl radical scavenging activity (HRSA) was calculated using the following equation: HRSA% = (A1− A2/A1) × 100. The measurements were performed four times.

### Cell culture

Murine 3 T3-L1 fibroblasts as preadipocytes were maintained in Dulbecco’s modified Eagle’s high-glucose medium (DMEM) containing 10 % calf serum, 100 units/mL penicillin, and 100 μg/mL streptomycin. Two-day post-confluent 3 T3-L1 cells, designated as day 0, were differentiated with complete DMEM containing 10 % FBS by adding a mixture (DMI) of 0.5 mM 3-isobutyl-1-methylxanthine, 100 μM indomethacin, 0.25 μM dexamethasone (DEX), and 167 nM insulin. The 3-isobutyl-1-methylxanthine, DEX, indomethacin, and Oil red O were purchased from Sigma-Aldrich (St. Louis, MO, USA). The medium was changed every 2 days. RCBs were added to the culture medium of the adipocytes on day 0. The cells were treated with 0, 50, or 150 μg/mL RCB extracts every day. After treatment with RCB on day 4 and day 7, the 3 T3-L1 adipocytes were harvested and lysed for Western blot analysis as described in section 2.12. To analyze cell viability, the cytotoxicity of the RCB was evaluated using 3-(4, 5-demethylthiazol-2-yl)-2, 5-diphenyltetrazolium bromide (MTT) according to the manufacturer’s instructions.

### Oil red O staining and triglyceride assay

Lipid accumulated within cells was visualized by Oil red O staining as described previously [27]. Oil red O staining was performed on day 7 of differentiation to stain accumulated lipid droplets in 3 T3-L1 adipocytes. The cells were gently washed with phosphate-buffered saline (PBS) and stained with filtered Oil red O solution (60 % isopropanol and 40 % water) for 30 min. After staining the lipid droplets red, the Oil Red O staining solution was removed, and the plates were rinsed with water and dried. The photographs were taken using an Olympus microscope (Tokyo, Japan). To analyze the content of cellular triglycerides, the cells were washed with PBS, scraped into 200 μL of PBS and sonicated for 1 min. The 3 T3-L1 cells were incubated with or without the selective PI3K inhibitor LY294002, which was purchased from Sigma-Aldrich (St. Louis, MO, USA) at a concentration of 10 μM in the presence or absence of RCB for 7 days. Total triglyceride content was measured in cell lysates using an assay kit, and cellular protein was measured using a Bio-Rad protein assay kit (Hercules, CA, USA). The results are expressed as percentage changes. All the experiments were carried out in triplicate.

### RT-PCR

Total RNA was isolated from the 3 T3-L1 adipocytes using Trizol reagent (Invitrogen, CA, USA) according to the manufacturer’s protocol. The total RNA (1 μg) was reverse-transcribed to cDNA using a reverse transcription system (Invitrogen, CA, USA) according to the manufacturer’s protocol. The mRNA expression of adipocyte differentiation-related genes was examined using cDNA and the following gene-specific primers: C/EBPβ, 5′-GACTACGCAACACACGTGTAACT-3′ and 5′-CAAAACCAAAAACATCAACAACCC-3′; PPARγ, 5′-TTTTCAAGGGTGCCAGTTTC-3′ and 5′-AATCCTTGGCCCTCTGAGAT-3′; C/EBPα, 5′-TTACAACAGGCCAGGTTTCC-3′ and 5′-GGCTGGCGACATACAGATCA-3′; β-actin (control), 5′-GACAACGGCTCCGGCATGTGCAAAG-3′ and 5′-TTCACGGTTGGCCTTAGGGTTCAG-3′.

### Western blot analysis

Western blotting was performed according to standard procedures [6] with slight modifications. Briefly, whole cells were lysed in lysis buffer containing 50 mM Tris–HCl (pH 8.0), 0.4 % Nonidet P-40, 120 mM NaCl, 1.5 mM MgCl_2_, 0.1 % SDS, 2 mM phenylmethylsulfonyl fluoride, 80 μg/ml leupeptin, 3 mM NaF and 1 mM DTT. Cell lysates were separated by 10 % SDS-polyacrylamide gel electrophoresis, transferred onto a polyvinylidene fluoride membrane (Amersham Pharmacia, England, UK), blocked with 5 % skim milk and hybridized with primary antibodies. The following antibodies were purchased from Cell Signaling technology (Danvers, MA USA): PPARγ, C/EBPβ, C/EBPα, aP2, FAS, Akt, and GSK3β; the monoclonal β-actin antibody was purchased from Chemicon (Millipore Inc., Billerica, MA USA). HRP-labeled mouse anti-rabbit IgG was obtained from Jackson ImmunoResearch. The Chemiluminescence kit was from Pierce (Rockford, IL). After incubation with horseradish-peroxidase-conjugated secondary antibody at room temperature, immunoreactive proteins were detected using a chemiluminescent ECL assay kit (Amersham Pharmacia, UK) according to the manufacturer’s instructions.

### Measurement of glucose uptake

Glucose uptake in the 3 T3-L1 cells was measured using the fluorescent D-glucose analogue 2-[N-(7-nitrobenz-2-oxa-1,3-diazol-4-yl)amino]-2-deoxy-D-glucose (2-NBDG) (Invitrogen, Carlsbad, USA) with slight modifications to a previously reported protocol [28]. Briefly, 3 T3-L1 pre-adipocytes were differentiated in various concentrations (0, 50, and 150 μg/ml) of RCB or DMSO for 7 days in 6-well plates. After washing, the differentiated 3 T3-L1 cells were treated with or without the indicated concentrations of RCB and insulin in the absence or presence of 10 μM 2-NBDG. After incubation for 2 h, the cells were washed three times with PBS, and the resulting fluorescence was measured (excitation at 485 nm and emission at 530 nm) using a fluorescent microplate reader (Molecular Devices, USA). All the experiments were carried out in triplicate.

### Animal experiments

Five-week-old Sprague–Dawley male rats were obtained from Central Lab Animal Inc. (Seoul, Korea). All animal experiments were conducted in accordance with the National Institutes of Health (NIH) guidelines and with the approval of the Animal Care and Use committee of Gyeongsang National University (Approval Number: GNU-140513-R0049). The rats were housed in polycarbonate cages in a room maintained at 22 °C with 55 % relative humidity on a 12 h dark/light cycle. All of the rats were allowed free access to food and water for five weeks. The rats were randomly divided into three groups that were fed a normal diet (ND, *n =* 10), a high-fat diet (HFD, *n =* 10), or a HFD supplemented with RCB (HFD + RCB, *n =* 10) for 5 weeks. The ND group was maintained on a normal diet based on a commercial diet (#55VXT0038, Samyang Co, Korea). The HFD group was fed an HFD based on a commercial diet (rodent diet with 60 % kcal fat, Research Diet, Korea). To test the anti-obesity activity of RCB extracts (200 mg/kg BW) orally administered to the HFD-fed rats for five weeks. Food intake was measured daily, and body weight was measured every two days. Rats were food deprived overnight prior to blood collection and euthanized by ketamine (40 mg/kg) plus xylazine (2 mg/kg).

Group 1: Normal diet group (ND)

Group 2: High-fat diet group (HFD)

Group 3: HFD + RCB 200 mg/kg BW (HFD + RCB)

### Biochemical and histological analysis

Body weight and fatty tissue mass (*n =* 10 rats/group) were measured with sensitivity limits of 0.1 g and 0.01 g, respectively. Blood samples were collected and centrifuged 1000 x g for 15 min at 4 °C, and serum was separated to analyze plasma biomarkers. Epididymal fat pads were excised, weighed and stored at −20 °C until they were assayed. Concentrations of plasma triglyceride (TG), total cholesterol (TC), and high-density lipoprotein-cholesterol (HDL-C) were assayed enzymatically using commercial kits (Asan phams, Co., Korea) as previously described [27]. There were 10 animals per group in all experiments. Epididymal adipose tissue samples were dehydrated, embedded in paraffin, cut into 5-μm sections and stained with hematoxylin-eosin for microscopic assessment (Olympus, Tokyo, Japan). For quantification analysis, adipocyte dimensions were measured using MetaMorph image analysis software (Molecular devices, CA, USA) equipped for the automation and programmable distance and color segmentation (*n =* 5 rats/group).

### Statistical analysis

Data were analyzed using SPSS version 12.0 software (SPSS Inc., Chicago, IL, USA). Each experiment was performed in at least triplicate. The data are expressed as the mean ± SD. Differences among multiple groups were analyzed by conducting one-way analysis of variance (ANOVA) followed by Duncan’s multiple range test, and differences between 2 groups were identified using Student’s *t* test. *P <* 0.05 was considered statistically significant and extremely significant between groups.

## Results

### Total phenol content (TPC) and total flavonoid content (TFC) of RCB

The antioxidant properties of RCB extracts are summarized in Table 1. The TPC and TFC content of RCB extract was found to be 125.3 ± 17.56 catechin equivalents/g and 35.45 ± 3.65 quercetin equivalents/g of extract, respectively (Table 1). RCB showed strong DPPH radical scavenging activity. The hydroxyl radical scavenging activity of RCB was measured by the inhibition of nitroblue tetrazolium and by RCB’s reduction of hydroxyl radical generation. The RCB extract also exhibited a potent scavenging effect on superoxide radicals.

**Table 1:** Radical scavenging activities, total phenolic and flavonoid contents of RCB extract

	DRSA	HRSA	SRSA	TP (mgCE/g)	Flavonoids (mgQE/g)
RCB	28.24 ± 2.05^a^	13.38 ± 1.47^a^	32.43 ± 2.40^b^	125.30 ± 17.56	35.45 ± 3.65
BHT	42.73 ± 0.50^b^	13.76 ± 2.01^a^	26.48 ± 1.95^a^	-	-
Ascorbic acid	75.42 ± 3.33^c^	18.14 ± 1.73^b^	24.27 ± 2.17^a^	-	-

DRSA, DPPH (2,2-diphenyl-1-picrylhydrazyl) radical scavenging activity; HRSA, hydroxyl radial scavenging activity; SRSA, superoxide anion radical scavenging activity; TPC, total phenolic acid. Total phenolic acid is expressed as milligrams of catechin equivalents (CE)/g of extract, and total flavonoid content is expressed as milligrams of quercetin equivalents (QE)/g of extract. Butylated hydroxytoluene (BHT) and ascorbic acid at the concentration of 100 μg/mL were used as positive control. Each value represents mean ± SD (*n =* 4). ^a-c^Means with different superscript letters in the same column were significantly different by Duncan’s multiple range test (*p <* 0.05)

### Inhibitory effect of RCB on 3 T3-L1 pre-adipocyte differentiation

**Fig. 1:**
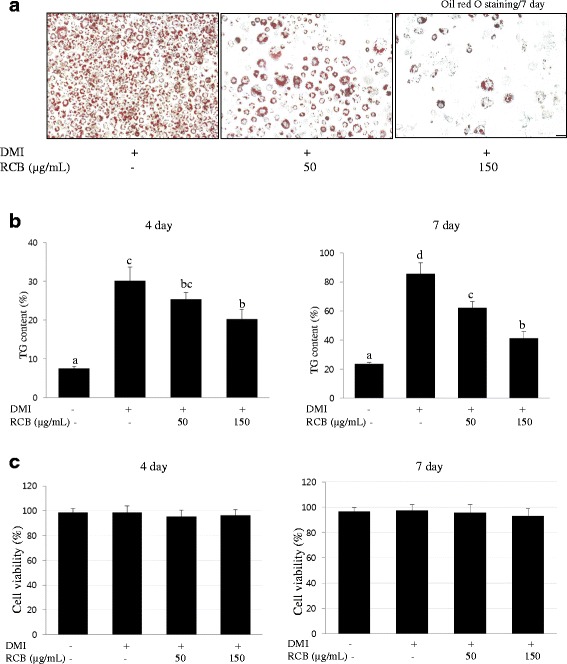
Effect of RCB on adipocyte differentiation in 3 T3-L1 cells. **a** Lipid accumulation in 3 T3-L1 adipocytes assessed by Oil red O staining. 3 T3-L1 pre-adipocytes were differentiated into mature adipocytes and then treated with RCB for 7 days. At day 7 post-induction, the cells were fixed, and neutral lipids were stained with Oil red O. DMI, fully differentiated adipocytes (0.5 mM 3 IBMX, 100 μM indomethason, 0.25 μM dexamethasone and 167 nM insulin); 50, fully differentiated adipocytes (DMI + 50 μg/ml RCB); 150, fully differentiated adipocytes (DMI + 150 μg/ml RCB). RCB represents *Rubus crataegifolius Bunge* extracts. The scale bar is 50 μm. **b** Effect of RCB on TG accumulation in differentiating 3 T3-L1 cells. Triglyceride content was measured using a triglyceride assay kit. The results shown are representative of at least three independent experiments. The values are presented as the means ± SD. Different letters on the each bar graph indicate that the differences were statically significant (I 0.05) according to Duncan’s multiple range test. **c** Effect of RCB on cytotoxicity during 3T3L1 pre-adipocyte differentiation into adipocytes. 3 T3-L1 cells were treated with RCB at various concentrations (0, 50, or 150 μg/ml) in a DMI mixture for 4 or 7 days. Cell viability after treatment with RCB was determined with an MTT assay

To examine the effects of RCB on the differentiation of pre-adipocytes into adipocytes, confluent 3 T3-L1 pre-adipocytes were treated with various concentrations (0, 50, and 150 μg/ml) of RCB extract in the presence or absence of a DMI mixture. The cell culture media was supplemented with RCB extract from day 0 to day 7, and the fully differentiated cells exhibited numerous lipid droplets, indicating lipid accumulation. On the seventh day of incubation, lipid accumulation was examined as a marker of differentiation by Oil red O staining. RCB extract showed anti-adipogenic properties, as indicated by the decreased levels of Oil red O staining on differentiation day 7 in Fig. 1a. To further investigate whether RCB had an effect on adipocyte differentiation, lipid accumulation was quantified by measuring triglyceride content during 3 T3-L1 differentiation. The triglyceride content of the cells increased during 3 T3-L1 differentiation over the course of 7 days, whereas the addition of RCB extract into the differentiation media strongly blocked triglyceride accumulation (Fig. 1b). These results showed that treatment with 50 or 150 μg/ml RCB extract resulted in a 17 % or 28 % decrease, respectively, in triglyceride accumulation in the 3 T3-L1 adipocytes.

### Cytotoxic effects of RCB on 3 T3-L1 cells

The toxic concentration of the RCB extract was assessed via an MTT viability assay using thiazoyl blue tetrazolium bromide. 3 T3-L1 cells were treated with different concentrations of RCB (0, 50, and 150 μg/ml) in combination with a DMI mixture for either 4 or 7 days. There were no significant differences in 3 T3-L1 cell viability at concentrations of up to 150 μg/ml of RCB extract compared to the control (Fig. 1c). These results demonstrated that RCB strongly blocks adipocyte differentiation in 3 T3-L1 cells.

### Inhibitory effect of RCB on adipogenic-specific gene and lipogenic gene expression

**Fig. 2:**
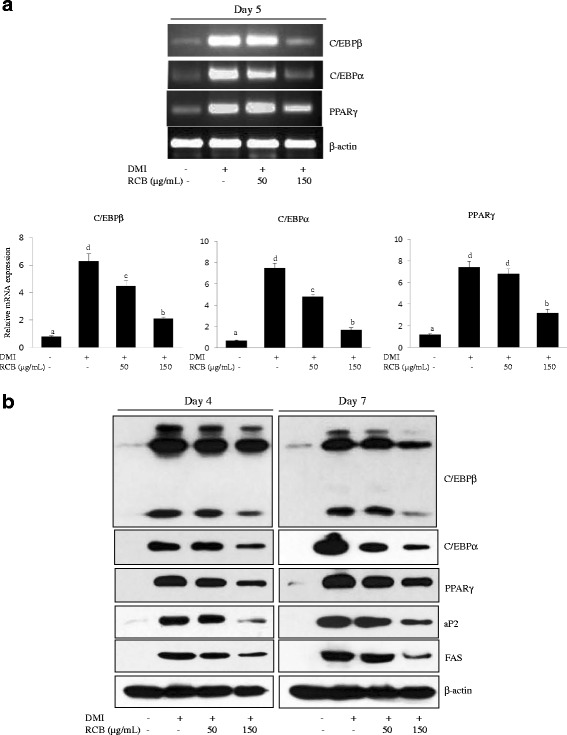
Effects of RCB on the mRNA and protein expression of genes related to adipogenesis. **a** RCB inhibited the mRNA expression of adipocyte-specific factors during 3 T3-L1 adipocyte differentiation. Differentiation of 3 T3-L1 pre-adipocytes into adipocytes was induced using DMI media in the absence or presence of RCB (50 μg/mL or 150 μg/mL) for 5 days. β-actin expression in each sample was used as an internal control to normalize expression. The results shown are representative of at least three independent experiments. The different letters on each bar graph indicate significant differences (*p <* 0.05), as determined by Duncan’s multiple range test. **b** RCB inhibited the protein expression of adipocyte-related factors in 3 T3-L1 adipocytes. Cell lysates from the 3 T3-L1 cells were prepared at day 4 or day 7 after the induction of differentiation. Western blotting analysis was described in detail in the *Materials and Methods* section

Adipocyte differentiation is accompanied by changes in the expression of various adipogenesis- and lipogenesis-related genes. C/EBPs and PPARγ are known adipogenic genes that have roles in transforming pre-adipocytes into mature adipocytes [5]. To investigate the anti-adipogenic mechanism of RCB, its effects on both mRNA and protein levels of C/EBPα, C/EBPβ, and PPARγ were elucidated. After inducing differentiation, 3 T3-L1 cells were exposed for 4 or 7 days to a concentration of 50 and 150 μg/ml RCB. As shown in Fig. 2a, the mRNA level of C/EBPβ was significantly lower in cells treated with 150 μg/ml RCB extract during adipocyte differentiation than that in the cells treated with DMI alone. The expression of C/EBPα and PPARγ mRNA was also significantly reduced following treatment with RCB extract (Fig. 2a). Furthermore, the protein levels of C/EBPβ, C/EBPα, and PPARγ were reduced in a dose-dependent manner after RCB treatment for 4 or 7 days (Fig. 2b). The activation of C/EBPα and PPARγ induces the expression of lipogenesis-related genes, including aP2 and FAS [5]. Thus, we examined whether the above-detailed down-regulation was related to decreased C/EBPα and PPARγ target gene expression levels. Consistent with the above observations, RCB suppressed the expression levels of C/EBPα and PPARγ target genes, such as aP2 and FAS (Fig. 2b). We also found that RCB extract treatment resulted in a dose-dependent suppression of aP2 and FAS at the protein level. Taken together, these results suggest that the activation of C/EBPα and PPARγ plays a critical role in the regulation of adipogenesis by RCB.

### Effect of RCB on the phosphorylation of Akt and GSK3β during adipocyte differentiation

**Fig. 3:**
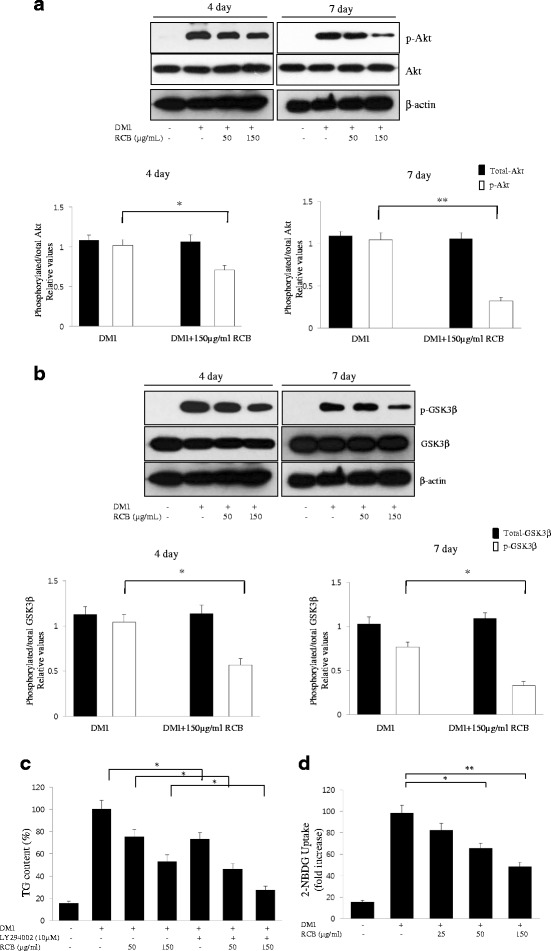
Effect of RCB on Akt and GSK3β phosphorylation during 3 T3-L1 adipogenesis. **a** RCB downregulated Akt phosphorylation induced by DMI in 3 T3-L1 adipocytes. The 3 T3-L1 pre-adipocytes were induced to differentiate with DMI media in the absence or presence of RCB for 4 or 7 days, and the phosphorylation levels of Akt were detected with its specific antibody. The results are reported as the means ± SD of three independent experiments. *P < 0.05. **P < 0.01. **b** Effect of RCB on GSK3β phosphorylation during 3 T3-L1 adipogenesis. The phosphorylation levels of GSK3β were determined using a specific antibody. The results are reported as the means ± SD of three independent experiments. *P < 0.05. **c** Inhibitory effects of LY294002 on 3 T3-L1 adipocyte differentiation. 3 T3-L1 cells were incubated with or without RCB at a concentration of 50 or 150 μg/ml during differentiation in the presence or absence of LY294002 (10 μM). After differentiation, the TG contents in the 3 T3-L1 adipocytes were determined by a triglyceride assay. The data presented are the mean ± SD from three independent experiments. *P < 0.05. **d** RCB inhibited DMI-stimulated glucose uptake in 3 T3-L1 adipocytes. Glucose uptake activity in differentiated 3 T3-L1 cells was analyzed by measuring the fluorescence intensity of the cells after incubation with 10 μM 2-NBDG for 2 h. The results are reported as the mean ± SD of three independent experiments. *P < 0.05. **P < 0.01

To investigate whether Akt, a major factor in adipocyte differentiation, is regulated by RCB during 3 T3-L1 differentiation, levels of phosphorylated Akt and GSK3β were analyzed and compared with total levels of Akt. Our results showed that DMI-stimulated 3 T3-L1 adipocytes exhibited strongly increased phosphorylation levels of Akt (Ser473) and GSK3β (Ser9). The phosphorylation of Akt was significantly decreased in the RCB-treated groups compared to the differentiated 3 T3-L1 adipocytes (Fig. 3a). Likewise, the expression of phospho-GSK3β was also substantially decreased following treatment with RCB during 3 T3-L1 cell differentiation (Fig. 3b). These results suggest that RCB extract treatment decreases Akt phosphorylation and down-regulates the phosphorylation of GSK3β, a substrate kinase of Akt, leading to the inhibition of the adipogenesis pathway.

### RCB inhibited 3 T3-L1 adipocyte differentiation through the Akt signaling pathway

To further understand the underlying mechanism of the inhibitory effects of RCB on adipocyte differentiation, we performed an experiment with LY294002, a chemical inhibitor of the Akt pathway. After treatment with DMI for 7 days, differentiated 3 T3-L1 cells showed increased accumulation of triglyceride droplets compared to undifferentiated 3 T3-L1 cells (Fig. 3c). Interestingly, treatment with 10 μM LY294002 resulted in a decrease in intracellular triglyceride accumulation in the differentiated 3 T3-L1 cells. Moreover, we found that the inhibitory effects of RCB on the formation of lipid droplets were more enhanced by the combined treatment of LY29400 and RCB than by treatment with RCB alone (Fig. 3c). These results indicate that the role of Akt signaling is significantly associated with the functional modulation of the RCB, which reduces adipogenesis in 3 T3-L1 cells.

### RCB reduced glucose uptake in 3 T3-L1 adipocytes

To examine the effects of RCB on glucose uptake in 3 T3-L1 adipocytes, a fluorescent deoxyglucose analog (2-NBDG) was used to calculate glucose uptake rates. 3 T3-L1 cells were exposed to 0, 10, 50, and 150 μg/ml concentrations of RCB extract in differentiation media for 7 days. The RCB treatment led to a significant dose-dependent decrease in glucose uptake compared to cells treated with DMI alone; glucose uptake in the 50 and 150 μg/ml RCB-treated groups showed decreases of 27 % and 38 %, respectively, compared to the DMI alone group (Fig. 3d).

### RCB induced decreases in body weight and adipose tissue weight in HFD-induced obese rats

**Fig. 4:**
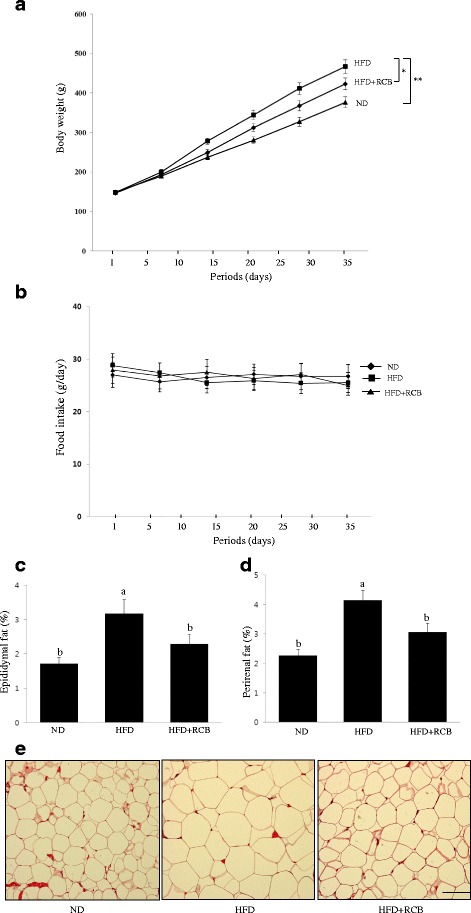
Effects of RCB extracts on body weight in HFD-induced obese rats. **a** Body weight gain. The rats were divided into three groups (*n =* 10): an ND group given a normal diet (ND), an HFD group fed an HFD, and an HFD + RCB group fed an HFD in addition to treatment with RCB (200 mg/kg BW) orally by gavage once a day for 5 weeks. There were significant differences between the body weights of the HFD and ND (**P < 0.01) and HFD and HFD + RCB groups (*P < 0.05) at the end of the experimental period. **b** Food intake. The mean daily food consumption was 26.7 g and no significant differences were found in food intake between ND and HFD groups. **c** Adipose tissue mass. The weight of the epididymal adipose tissue was measured by dividing fatty tissue weight by body weight (fatty tissue/body weight x 100). Values represent the means ± SD; *P <* 0.05, as shown by ANOVA. Bars labeled with different letters indicate significant differences at according to Duncan’s multiple range test. **d** The weight of the perirenal adipose tissue was measured by dividing fatty tissue weight by body weight (fatty tissue/body weight x 100). The data presented are the mean ± SD from three independent experiments. Values represent the means ± SD; *P <* 0.05 as shown by ANOVA. Different letters (**a** and **b**) mean that values are significantly different among groups. **e** Representative hematoxylin and eosin-stained sections of epididymal adipose tissue. The adipocyte sizes from the HFD + RCB group were smaller than those from the HFD only group. The scale bar is 100 μm

We further evaluated whether RCB possessed anti-obesity effects in rats fed a HFD for 5 weeks. At the end of the experiment, the body weights of the HFD group were 39 % greater than those of the ND control group, demonstrating that HFD-fed rats developed obesity (Additional file 1 and Fig. 4a). The weight gain observed for the group of rats that were administered RCB extract (200 mg/kg/daily) was markedly decreased compared to the HFD only group. We did not observe any significant changes in food intake in any of the treatment groups compared to the HFD-fed group and the HFD-fed plus RCB extract treatment group (data not shown). Thus, these data indicate that the reduction in body weight gain in the RCB-treated rats was not mediated by a reduction of food and water intake in the HFD rats. To investigate whether the reduction in body weight gain in the RCB-treated group was related to decreased fat accumulation, epididymal and perirenal adipose tissues were weighed. Supplementation with RCB significantly reduced epididymal fat (25 %) and perirenal fat (22 %) mass compared to the untreated HFD group (Fig. 4b and c). Histological examination of the epididymal adipose tissue revealed that adipocyte size was significantly smaller in the epididymal adipose tissue of the RCB-treated group than in that of the HFD only group (Fig. 4d), further demonstrating that the reduction in body weight gain primarily arose because of decreased fat accumulation and adipocyte size.

### RCB changed serum triglyceride (TG), total cholesterol and HDL cholesterol levels in HFD-induced obese rats

**Fig. 5:**
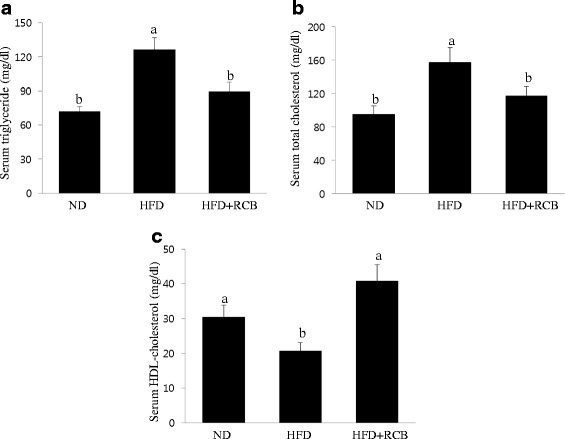
Effect of RCB on lipid content in HFD-induced obese rats. **a** Serum triglyceride level. The RCB extract-administered group showed significantly lower levels of serum triglyceride compared with the HFD group. Data are expressed as the means ± SD. Differences among multiple groups were analyzed with ANOVA followed by Duncan’s multiple range test. Different letters (**a** and **b**) indicate that the column means are significantly different at *P <* 0.05. **b** Total cholesterol level. Total cholesterol level decreased in the RCB-treated groups compared to the HFD only group. The results are presented as the means ± SD. Different letters (**a** and **b**) indicate that the column means are significantly different; *p <* 0.05 according to Duncan’s multiple range test. **c** HDL cholesterol level. A significant difference was observed in HDL cholesterol levels between the HFD and HFD + RCB groups. The values are expressed as the means ± SD. Different letters (**a** and **b**) on the each bar graph mean that values are significantly different among groups; *p <* 0.05 according to Duncan’s multiple range test

To support biochemical evidence underlying changes in epididymal and perirenal adipose tissues following treatment with RCB extract, we further investigated the effects of RCB on the serum levels of TG, total cholesterol (TC), and HDL-cholesterol (HDL-C) in HFD-induced obese rats. The serum levels of TG and TC were significantly reduced in the RCB extract-treated obese rats. Compared to the ND group, the serum levels of TG and TC in the HFD group increased, whereas the serum levels of TG and TC were significantly reduced by 29 % and 26 %, respectively, in the RCB extract-treated obese rats compared to the levels observed in the HFD only group (Fig. 5a and b). The serum HDL-C level was significantly higher in the HFD + RCB group compared to the HFD group (Fig. 5c).

## Discussion

Metabolic syndrome has become a major public health problem throughout the world. It is characterized by a cluster of risk factors that occur simultaneously, including insulin resistance, obesity, hypertension, and dyslipidemia, all of which considerably increase the risk of developing cardiovascular disease and type 2 diabetes mellitus [29]. Among these risk factors, obesity correlates most strongly with the prevalence of metabolic syndrome [30]. Due to the severe side effects of anti-obesity drugs, natural products for treating obesity have been recognized to be beneficial as an alternative.

In the present study, we investigated the anti-adipogenic effects of RCB in 3 T3-L1 adipocytes and evaluated its anti-obesity properties in HFD-induced obese rats. RCB extract treatment decreased lipid droplet formation in 3 T3-L1 adipocytes and reduced triglyceride accumulation in RCB-treated 3 T3-L1 adipocytes when used at a concentration of 150 μg/ml; these effects were not due to cellular cytotoxicity. These results demonstrate that RCB inhibits the differentiation of 3 T3-L1 preadipocytes into adipocytes as well as the accumulation of lipid droplets in cytoplasm, which is an adipocyte phenotype that occurs following differentiation based on lipid accumulation.

Differentiation-associated lipid accumulation was accompanied by induction of the adipogenic transcription factors C/EBPα and PPARγ, which are active during the early stages of adipocyte differentiation and stimulate the expression of numerous metabolic genes to produce an adipocyte phenotype [31]. C/EBPα induces the autoactivation of its own transcription as well as PPARγ expression, and it maintains the expression levels of PPARγ and C/EBPα, which enables PPARγ to stimulate adipocyte differentiation. Rosen et al. [32] reported that C/EBPα supports adipocyte-specific gene expression in the presence of PPARγ in genes associated with cell morphology and lipid accumulation. In the present study, we found that treatment of 3 T3-L1 adipocytes with RCB extract exhibited decreased mRNA expression and protein expression of C/EBPα and PPARγ, which are master regulators of adipocyte differentiation. Moreover, our data indicated that the exposure of 3 T3-L1 adipocytes to RCB extract significantly decreased the expression levels of aP2 and FAS compared to differentiated 3 T3-L1 adipocyte cells. aP2, a member of the cytoplasmic fatty acid-binding protein family, has been detected in adipose tissue, and its expression is highly regulated during the differentiation of adipocytes [33]. FAS is a lipogenic enzyme that stimulates triglyceride synthesis and facilitates fatty acid storage in the cell cytoplasm [34]. PPARγ activation is required for proper functioning of the fat-selective enhancement of the aP2 and FAS genes in adipocytes [5]. Overall, we suggested that RCB inhibited adipocyte differentiation by reducing the expression of adipocyte-specific factors, including C/EBPβ, C/EBPα, and PPARγ, which leads to the downregulation of lipogenesis-related genes, such as aP2 and FAS.

Activation of the Akt pathway during adipogenesis has been shown to promote differentiation by activating factors that regulate the C/EBP family and PPARγ expression [9]. Insulin signaling activates Akt through PI3K and induces the serine/threonine phosphorylation of downstream targets, such as GSK3β [35]. Expression of a constitutively active variant of Akt kinase in 3 T3-L1 cells has been shown to result in the spontaneous differentiation of fibroblasts into adipocytes, which is associated with the increased accumulation of lipid droplets [13]. A previous study used LY294002 to demonstrate that the function of PI3-kinase was indispensable to lipid accumulation in 3 T3-L1 adipocytes. Thus, the PI3-kinase and Akt kinase pathways play pivotal roles in terminal adipocyte differentiation. Therefore, to explore the above-mentioned question, we examined whether RCB increased TG accumulation and the expression of adipocyte-related genes through the Akt signaling pathway.

In the present study, we showed that RCB caused a marked attenuation in Akt phosphorylation induced by insulin in a dose-dependent manner. RCB extract treatment also strongly suppressed insulin-induced GSK3β phosphorylation in 3 T3-L1 adipocytes. DMI induced a strong increase in the Akt phosphorylation in 3 T3-L1 cells, which is consistent with the observed enhancement in Akt activation. Moreover, treatment of 3 T3-L1 cells with a combination of LY294002 and RCB showed significantly stronger inhibitory effects on triglyceride accumulation than treatment with RCB alone. In addition, our present results confirmed that the level of Akt phosphorylation decreased significantly after RCB extract treatment, which also significantly reduced insulin-stimulated glucose uptake, indicating that the Akt signaling pathway might be involved in the inhibitory effect of RCB on 3 T3-L1 adipocyte differentiation. Based on these findings, we concluded that RCB acts through Akt kinase pathways, resulting in decreased levels of TG accumulation and adipocyte-related gene expression. Therefore, our results suggest that the inhibition of Akt phosphorylation and activation via RCB blocked insulin-induced adipocyte differentiation in 3 T3-L1 pre-adipocytes.

Previous studies have revealed that the fruits of raspberry species have essential positive effects on the human diet and human health, which are likely mainly due to their medicinally active phytochemicals, such as polyphenols, various flavonoids (such as anthocyanins and flavanols), condensed and hydrolysable tannins and phenolic acid derivatives [36]. The major polyphenols in raspberries are anthocyanins and ellagitannins [37, 38], which comprise > 90 % of the total phenolic content of the fruit. Many studies have shown that the polyphenols present in green tea, Schisandra chinensis, blueberry peel, and grapefruit combat adipogenesis at the molecular level and also induce lipolysis [27, 39]. The administration of *Rubus coreanus* (RC) to HFD-fed mice combined with exercise has previously been shown to accelerate energy expenditure and protect against oxidative stress [40]. RC exerts an anti-obesity effect by upregulating Carnitine palmitoyl transferase I (CPT1) and elevating antioxidant levels [40]. In the present study, our data revealed that RCB exhibited free radical scavenging activity similar to DPPH, superoxide anion and hydroxyl radicals, and displayed potent antioxidant activity correlated with levels of strong phenols and flavonoids.

Adipose mass, which reflects the average adipocyte size and total adipocyte number, is increased in obese individuals. Adipogenesis occurs when extra fat mass is required for caloric gain or for cellular homeostasis [41]. Increased sizes (hypertrophy) and numbers (hyperplasia) of fat cells were present in the HFD-induced obese rats in the current study. Adipose tissue hyperplasia is a result of increased adipogenesis, which includes pre-adipocyte proliferation and adipocyte differentiation. We investigated whether RCB modulates obesity in HFD-induced obese rats. These results showed that the body weights of rats that were fed a HFD plus RCB extract were significantly reduced and comparable to those of rats that were fed a control HFD diet.

To examine whether the reduction in body weight gain exhibited by the RCB-treated group was related to decreased fat accumulation, epididymal and perirenal adipose tissues were weighed. The weights of both of these tissue types were significantly reduced in the RCB-treated group compared to the HFD group, indicating that the alleviation of obesity in the RCB-treated rats was due to reduced adiposity in adipose tissues. In addition to the effects of RCB on epididymal fat mass, histological examinations of the epididymal adipose tissues revealed that RCB extract greatly decreased the average size of adipocytes in the HFD-induced obese rats. The adipocyte size in epididymal adipose tissue was 26 % lower in the HFD-RCB group compared to the adipocyte size in the HFD group, which further demonstrated that the reduction in body weight gain was primarily due to decreased fat accumulation in adipocytes.

Excessive body weight gain causes obesity-related health problems and dyslipidemia, characterized by an increase in circulating triglycerides and a decrease in high-density lipoprotein cholesterol (HDL-C) [42, 43]. In particular, triglyceride accumulation is mainly responsible for weight gain or adiposity, accelerating the development of obesity and metabolic diseases. In parallel with its effect on body weight, we also found that RCB extract supplementation significantly decreased serum TG and TC levels and increased serum HDL-C level, indicating that RCB efficiently prevents abnormal metabolism of TC and cholesterol in HFD-induced obese rats. Therefore, our results demonstrate that the effects of RCB supplementation were much more evident with respect to reductions of adipose tissue mass compared to body weight gain. Overall, these results may shed light on the fact that RCB suppressed HFD-induced increases in adipose tissue mass and body weight gain, which may reflect the influence of adipose tissue metabolism, particularly by increasing lipid excretion.

## Conclusion

In the present study, we investigated the anti-obesity effects of RCB on adipocyte differentiation and the associated mechanisms in 3 T3-L1 cells, and we confirmed our findings in a rat model of obesity that was induced with a HFD. Treatment with RCB extract significantly inhibited the expression of C/EBPβ and subsequently downregulated the activation of the key transcriptional regulators C/EBPα and PPARγ in 3 T3-L1 adipocytes. The phosphorylation levels of Akt and its substrate GSK3β were significantly reduced in response to RCB treatment, suggesting that RCB induced the inhibition of adipogenesis by regulating Akt phosphorylation and the upstream signaling of C/EBPα and PPARγ in 3 T3-L1 cells. Moreover, RCB significantly decreased body weight gain, body fat accumulation, and serum TG and TC levels in HFD-induced obese rats, which can prevent the development of HFD-induced obesity. Our results in cell lines and animal studies revealed that the anti-obesity effects of RCB were caused by decreased adipogenesis. It may be worthwhile to explore whether RCB could serve as a suitable candidate for enhancing anti-obesity activity or the development of therapeutic supplements for obesity.

## Additional file

Additional file 1: Effects of supplementing RCB on body weight gain, and serum profiles in rats fed a high fat diet for two weeks. (A) Weight gain, (B) serum triglyceride level, and (C) total cholesterol level. The values are expressed as the mean ± SD. The bars showing different letters indicate significant differences among each group of bars, according to Duncan’s test; **p <* 0.05. (ZIP 90 kb)

## Abbreviations

RCB: rubus crataegifolius bunge
FAS: fatty acid synthase
HFD: high-fat diet
C/EBPδ: CCAAT/enhancer binding protein-δ
C/EBPα: CCAAT/enhancer binding protein-α
PPARγ: peroxisome proliferator-activated receptor-γ
HDL-C: high-density lipoprotein-cholesterol
PI3K: phosphoinositide 3-kinase
TG: triglyceride
aP2: adipocyte fatty acid-binding protein 2
DPPH: 2,2-diphenyl-1-picrylhydrazyl
HRSA: hydroxyl radial scavenging activity
SRSA: superoxide anion radical scavenging activity
TPC: total phenolic acid
MIX: 3-isobutyl-1-methylxanthine
DEX: dexamethasone
TC: total cholesterol
HDL-C: high-density lipoprotein-cholesterol
FAS: fatty acid synthase CE, catechin equivalents
QE: quercetin equivalents
HPLC: high-performance liquid chromatography
NBT: nitroblue tetrazolium
CPT1: Carnitine palmitoyl transferase I
